# Determinants of the intention to donate umbilical cord blood in pregnant women

**DOI:** 10.1111/vox.13179

**Published:** 2021-07-14

**Authors:** Mariana Fernandes, Guido Alessandri, Rubi Abbad, Caterina Grano

**Affiliations:** ^1^ Department of Psychology Sapienza University of Rome Rome Italy

**Keywords:** donor motivation, donors, embryonic stem cell

## Abstract

**Background and Objectives:**

Umbilical cord blood (UCB) donation is a behaviour promoted by many countries' health systems. However, UCB donation is not a widespread behaviour among expectant mothers, and little is known about the reasons that may lead to it. The aim of the present study was to analyse the contribution of Theory of Planned Behaviour (TPB) variables among both primiparous and multiparous women in predicting intention to donate UCB.

**Materials and Methods:**

Three hundred seventy‐six expectant mothers completed questionnaires that captured sociodemographic data, parity, previous donation, attitudes, subjective norms, perceived behavioural control (PBC) and intention to donate UCB. Multigroup analysis structural equation modelling was conducted using Mplus (version 8.02).

**Results:**

Multigroup path analyses showed that intentions were strongly predicted by subjective norms and moderately predicted by positive attitudes and PBC in both primiparous and multiparous women. TPB constructs explained 71% of the variance in intentions for both groups.

**Conclusions:**

Future interventions to increase intention to donate among primiparous and multiparous women could primarily consider the influence of partner and significant others in determining positive intentions and secondarily target increasing positive attitudes and perceptions of control.

## INTRODUCTION

Umbilical cord blood (UCB) banking consists in the collection and storage of the UCB from the placenta and umbilical cord, soon after childbirth [1] to preserve potentially life‐saving cells that are usually considered medical waste [2, 3]. Cord blood transplantation is considered a valid alternative treatment in haematologic diseases (malignant and non‐malignant), primary immunodeficiencies and metabolic disorders [4, 5], especially in the case of scarce probability of having a compatible donor [5]. Moreover, clinical studies have proved the pluripotent nature of cord blood cells [6, 7], highlighting a wide range of possible clinical applications in neonatology [6, 8], regenerative medicine and immune modulation [9]. UCB can thus be used in the treatment of various life‐threatening diseases such as leukaemia, cerebral palsy, neonatal hypoxic–ischaemic encephalopathy and diabetes.

UCB can be stored either in a public bank for allogeneic use (available to anyone who might be compatible) or in private banks for autologous or family use [1]. Several European and US professional organizations and institutions [10] support and promote the donation of UCB to public institutions for altruistic and solidarity purposes (e.g., patients needing transplantation will be able to rapidly find a matching donor), since the use of cord blood stored in private cord blood banks for autologous use rarely occurs [11]. Although in the previous two decades, many public banks for allogeneic use have been established in Italy, actual donations remain very low, with only 2.2% of parents donating the cord blood to a public cord blood bank [12]. There are several possible reasons for the current low cord blood donation rates. For instance, although women and/or couples are aware of the possibility of donating UCB, they may either have little knowledge of donation possibilities or the procedures to be followed or even be unaware of the uses of UCB [13, 14]. In the view of these premises, it seems particularly relevant to understand the factors that influence expectant parents' decisions to donate cord blood, as this may be essential for encouraging donation.

Considering demographic factors, results of previous studies indicated that educational level and age are positively associated with UCB donation [5, 15, 16], while mixed results are reported on what concerns household income [4, 16].

Although a growing number of studies highlighted the role of psychosocial factors in explaining, predicting and promoting different donation behaviours [17, 18], research on UCB donation is still very limited with only a few studies highlighting a positive association between attitudes and UCB donation [19, 20].

One of the most frequently applied models for predicting donation behaviours and social behaviours, in general, is the Theory of Planned Behaviour (TPB) [21]. In the TPB model, the most proximal determinant of behaviour is considered intention to perform that behaviour [21]. In turn, intentions are determined by attitudes (i.e., an evaluation or appraisal of the behaviour as favourable or unfavourable), subjective norms (i.e., perceived social pressure and expectations from significant others to perform the behaviour) and perceived behavioural control (PBC) (i.e., how much control individuals believe they have over the performance of the behaviour) [21, 22].

Considering the TPB in the more general context of blood donation, a systematic review reported that the model explained between 31% and 72% of the variance in blood donation intentions and between 54% and 56% in blood donation behaviour [17]. Across the studies, attitudes towards blood donation, subjective norms and PBC significantly predicted intention to donate blood, being the influence of subjective norms on intentions less consistent [17]. Furthermore, PBC had the strongest effect in predicting intentions to donate blood [17]. Similarly, in a meta‐analytic review [18] including 37 studies on blood donation intentions and 24 predictive studies on blood donation behaviour, intentions had the strongest associations with donation behaviour. In addition, large positive effects were found for attitudes and PBC in predicting intentions, while a medium positive association was observed for subjective norms [18]. Past donations were also positively associated with both intention to donate and donation behaviour [18].

Despite the successful use of the TPB in the context of blood donation [17, 23], to the best of our knowledge, only one study investigated the prediction of cord blood donation within this framework [24]. In this study, attitudes, normative beliefs, subjective norms, awareness and behaviour control were all considered predictors of women's intention to donate cord blood, explaining together 52% of the variance for intention to donate. However, it is not possible to infer the contribution of each of these constructs in this study, as the regression coefficients were not reported by the authors and the results were only summarily described [24]. Other studies have considered only some of the TPB variables [19, 20]. For example, Kim and colleagues [19] examined the knowledge and attitudes of early post‐partum women about storage, donation and disposal of their cord blood, as well as sociodemographic factors influencing cord blood donation, and found that knowledge, attitudes, income and source of information influenced cord blood donation. None of these studies has been conducted in the Italian context.

Based on these premises, the present study aims at extending previous research on the predictors of the intention to donate UCB considering primiparous and multiparous Italian women using the well‐established theoretical framework of the TPB.

## METHODS

### Participants

Three hundred seventy‐six pregnant women in the third trimester of pregnancy participated in the study. To be included, women had to be in the last semester of pregnancy and be fluent in Italian. Eleven participants with pathologies precluding the donation of UCB were excluded from the study. A total of 365 participants remained.

### Procedure

Expectant mothers were recruited through a variety of means (e.g., information centres for maternity and birth, family associations, cultural and sports associations). Participation in the study was anonymous and voluntary, and no incentives or payments were offered. The survey was administrated as an online questionnaire. First, participants read and signed a consent form and then were asked to provide their email address. Afterwards, participants received an email containing a personal code and the web link to access the online questionnaire, to which they had access only through that same personal code. The study was approved by the Ethical Committee of the Department of Psychology of ‘Sapienza’ University of Rome (Prot. 001066).

### Measures

The questionnaire, in addition to sociodemographic information (age, parity, gestation month, current relationship, education level of the expectant mother), included a question evaluating potential illnesses precluding UCB donation (e.g., autoimmune diseases, infectious diseases, etc.). Before starting to answer the questionnaire, women were asked if they were aware of the possibility of donating or conserving UCB. All these questions were answered on a dichotomous scale (yes/no).

### Theory of planned behaviour

Regarding the formulation and scaling of the TPB variables, we followed the questionnaire construction recommendations of Fishbein and Ajzen (2010) [25]. The items used to measure the TPB constructs are listed in Appendix S1.

Attitudes towards UCB donation were assessed using a combination of outcome beliefs measured by 10 semantic differentials: ‘Donate the blood cord of my baby would be for me…’ ‘useless–useful,’ ‘difficult–easy,’ ‘disappointing–rewarding,’ ‘senseless–sensible,’ ‘disadvantageous–advantageous,’ ‘unsatisfactory–satisfactory,’ ‘wrong–right,’ ‘stupid–wise,’ ‘unnerving–reassuring’ and ‘expensive–economical.’ Items were scored on a 7‐point Likert scale. Cronbach's alpha resulted in 0.91.

Subjective norms regarding UCB donation were measured using a combination of normative belief measures and motivation to comply measures. Subjective norms were measured through five items rated on a 7‐point Likert scale, ranging from completely disagree (1) to completely agree (7). Example of items were as follows: ‘Most people who are important to me think that I should donate the cord blood of my baby’ and ‘My partner would approve….’ Higher scores correspond to higher perceived social approval. Cronbach's alpha resulted in 0.90.

PBC over UCB donation was measured using a combination of control belief measures and power items. PBC was assessed with three items: ‘Suppose you decide to donate your baby cord blood. How easy or difficult do you think it will be?’, ‘very difficult’ (1) to ‘very easy’ (7). ‘Deciding to donate the cord blood of your baby is…’ ‘not at all up to me’ (1) to ‘completely up to me’ (7) and ‘How much control do you feel you have over your decision to donate your baby cord blood?’ ‘not at all under my control’ (1) to ‘completely under my control’ (7). Items were scored on a 7‐point Likert scale with higher scores representing greater PBC over cord blood donation. Cronbach's alpha resulted in 0.95.

Mothers' intention to donate cord blood was measured through five items. An example of an item was ‘Do you intend to donate your baby cord blood?’ ‘I definitely do not’ (1) to ‘I definitely do’ (7). The remaining items asked participants how strongly they wanted to donate, how likely it was for them to though about donate and how much they would like to donate. Items were scored on a 7‐point Likert scale with higher scores indicating stronger intentions to donate. Cronbach's alpha resulted in 0.95.

Past donation/storage for primiparous/multiparous woman was evaluated through a dichotomous question (yes/no).

### Data analysis

Descriptive analysis was conducted with the statistical program SPSS for Windows version 25.0 (IBM Corp, Armonk, NY). Data are reported as mean ± standard deviation (SD). Categorical data are reported as counts and percentages. To estimate the hypothesized model, we used M*plus* (version 8.02, Muthén & Muthén, Los Angeles, CA) [26] with the Satorra and Bentler [27] scaled chi‐square statistic (SB𝜒^2^) and robust standard errors, which takes into account the non‐normal distribution of the data (Mplus estimator = MLM). According to a multifaceted approach to the assessment of model fit [28], the following criteria were employed to evaluate the goodness of tested models: the Satorra–Bentler *χ* ^*2*^ likelihood ratio statistic supplemented by the comparative fit index (CFI) and the root mean square error of approximation (RMSEA), with associated confidence intervals. The significance value of *χ* ^*2*^ is sensitive to large sample sizes and easily produces a statistically significant result [29]. Values of RMSEA < .08 and CFI > .90 were considered acceptable [30].

Multiple‐group structural equation modelling was used to investigate measurement invariance across the two groups (primiparous and multiparous) [26]. In our approach, the equivalence across the two groups was tested as follows: after evaluating the configural model, each level of invariance was evaluated separately for groups and then simultaneously. The complete sequence of models is shown in Table 3. Overall, configural, metric, scalar and strong invariance [31] were evaluated. The plausibility of the equality constraints implied by the different models was examined with the Satorra–Bentler SB*⊿χ* ^*2*^ difference test between nested models (i.e., the sex constrained model vs. the unconstrained model [32]).

## RESULTS

The final sample was composed of 365 participants, with a mean age of 31.67 (SD = 5.39; age range = 18–44). Primiparous constituted 75.3% (*n* = 275) of the sample. Sociodemographic characteristics for the entire sample are reported in Table 1. The vast majority of respondents reported being aware of the possibility of donating or conserving UCB (97.5%). Considering past UCB donation, only 2.7% had previously donated UCB (*n* = 10).

**TABLE 1:** Participants sociodemographic characteristics (*N* = 365)

	Primiparous		Multiparous	
	(*n* = 275)		(*n* = 90)	
	*N*	%	*N*	%
Months of gestation				
Seventh	85	30.9	18	20.0
Eighth	101	36.7	42	46.7
Ninth	88	32.0	28	31.1
Tenth	1	0.4	2	2.2
Education level				
Primary school	—	—	1	1.1
Middle school	30	10.9	17	18.9
Higher school	122	44.4	50	55.6
Degree	123	44.7	20	22.1
Missing	—	—	0.5	2.2
Current relationship				
Married	142	51.6	59	65.6
Cohabitant	119	43.3	30	33.3
Have a partner (not cohabitant)	14	5.1	1	1.1
Mean age (SD)	31.22	5.24	33.09	5.63

Abbreviation: SD, standard deviation.

### Correlations

Table 2 presents the descriptive statistics and the zero‐order correlations among the mean scores on the overall measures of attitudes, subjective norm, PBC, past behaviour and behavioural intention. All correlations resulted significant and in the expected direction, except for those involving past behaviour in the multiparous group. Indeed, past behaviour resulted uncorrelated with all of the variables included in the model. It was, thus, decided to remove this variable from all subsequent structural equation models.

**TABLE 2:** Zero‐order correlations among study variables

Multiparous	ATT	NS	PBC	INT	PB	Mean	SD
ATT	1					6.20	1.42
NS	0.56**	1				4.52	2.28
PBC	0.19*	0.30**	1			5.57	1.99
INT	0.52**	0.66**	0.44**	1		4.62	2.18
PB	0.05	0.05	−0.03	0.04	1	0.87	0.38

Primiparous	ATT	NS	PBC	INT	PB	Mean	SD
ATT	1					6.07	1.66
NS	0.66**	1				4.68	2.32
PBC	0.39**	0.45**	1			5.43	1.99
INT	0.69**	0.87**	0.52**	1		4.95	2.32
PB	—	—	—	—	—	—	

*Note*: **p < 0.01; *p < 0.05.

Abbreviations: ATT, attitudes; INT, behavioural intention; NS, subjective norms; PB, past behaviour; PBC, perceived behavioural control; SD, standard deviation.

### Structural equation analysis

**FIGURE 1:**
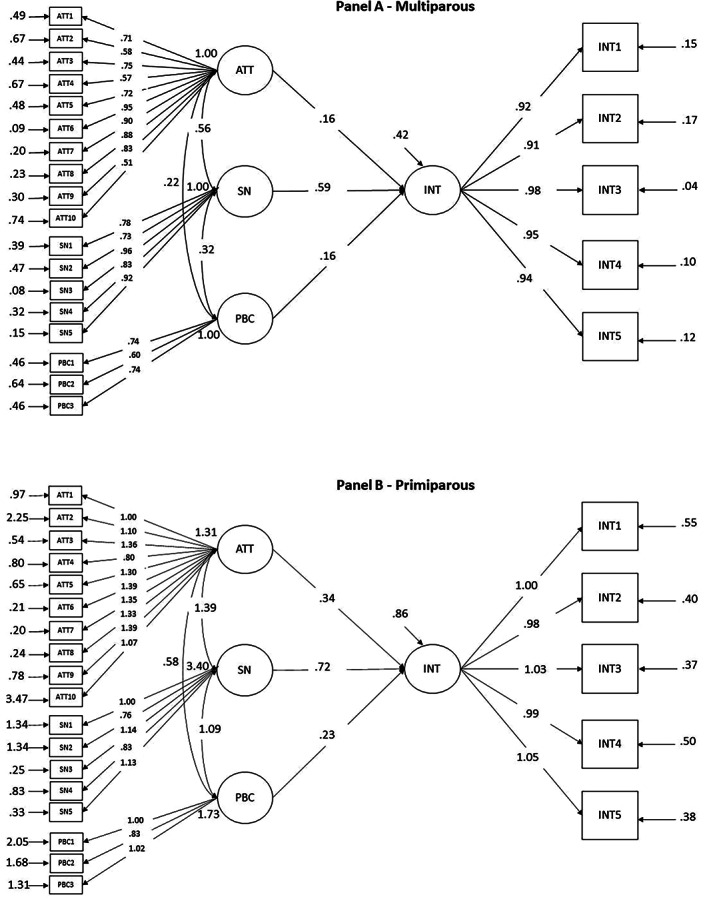
The hypothesized model with completely standardized estimates for (a) multiparous and (b) primiparous *Note*: ATT, attitudes; PBC, perceived behavioural control; INT, behavioural intention; SN, subjective norms

Our theoretical model was tested as a multiple‐group structural equation model simultaneously on primiparous and multiparous groups. In this model, attitudes, subjective norms, PBC and behavioural intention were specified as latent variables saturated, respectively, by 10, 5, 3 and 5 items. Parameters were all freely estimated across groups with no restriction imposed. As reported in Table 3, this model fitted the data very well. Following standard procedures, first, across‐group invariance was imposed on factor loadings and then on structural regression paths. Overall, these constraints did not change the fit of the model (See Table 3, bottom part). The final model with all restriction included is represented in Figure 1, panels (a) and (b). Overall, behavioural intention was strongly predicted by subjective norms, moderately predicted by attitudes and PBC. Subjective norms and attitudes were strongly correlated, whereas there were moderate correlations between subjective norms and PBC and between attitudes and PBC. Explained variance was high (R^2^ = 0.71 in both groups).

**TABLE 3:** Model fit and comparisons

Title	SB𝜒2	*scr*	*df*	CFI	RMSEA (95%CI)
M1. Unconstrained model	862.416	1.20	448	0.926	0.074 (0.067, 0.082)
M2. Equal loadings	881.717	1.21	467	0.926	0.073 (0.065, 0.080)
M4. Equal structural paths	885.133	1.21	470	0.926	0.073 (0.065, 0.080)

	SB*⊿*𝜒2		⊿*df*	*p*	
M2 versus M1	22.12		19	0.28	
M3 versus M2	3.42		3	0.31	

Abbreviations: CFI, comparative fit index; CI, confidence intervals; RMSEA, root mean square error of approximation.

## DISCUSSION

Stemming from the TPB, the aim of the present study was to explore the extent to which attitudes, subjective norms and PBC influence the intention to donate UCB in Italian primiparous and multiparous pregnant women [21].

For decades, TPB has been applied to effectively explain, predict and promote blood donation behaviour [17, 18], but, surprisingly, only one study used this framework to predict blood cord donation [24]. The present study findings extend previous research in the Italian context supporting the robustness of the TPB model in explaining blood cord donation intentions. The fit indices across the cross‐sectional analyses confirmed the model structure both in primiparous and multiparous women and the invariance across the two groups. Together, the variables included in the TPB model explained 71% of the variance in intentions to donate UCB in both samples, with subjective norms being the strongest determinant of intentions and attitudes and PBC significantly contributing to explaining intentions both in primiparous and multiparous women. This amount of explained variance is consistent with the results reported in other studies on blood donation [17]. Unfortunately, regarding cord blood donation in specific, further comparisons are limited, since Natan's study [24] suffers from a few methodological issues, namely because items were ill‐defined and regression results were only partially reported.

Differently from previous studies on blood donation [17, 18], it seems that subjective norms play a stronger role in determining intentions in the present study. The direction of the path from subjective norms to intention was positive, implying that higher perceived social approval for cord blood donation results in higher intention for UCB donation. This finding is not surprising considering that blood cord donation is generally a shared decision within a couple and that subjective norms also encompassed a partners' approval measure. In fact, previous studies showed that pregnant women consider their husband/spouse to have a key role in the decision about cord blood donation [4, 33]. Based on these findings, it would be desirable that future studies should also include the perspective of the partner and partner–actor interactions in determining blood cord donation intentions. Other explanations for this finding are also possible, such as the fact that although cord blood donation is well known, the women underline the need of knowing more clear procedures on cord blood banking processes [34] and turn to relevant others to form their intentions. Moreover, consistently with other studies on blood donation [17, 18], attitudes and PBC were also found to have a predictive role on intended behaviour donation.

Some practical implications can be drawn based on the present findings. Interventions directed towards raising UCB donation intentions may need to target relevant others (partner, families and health professionals) as their opinion may influence expectant mothers intentions. Interventions should also consider increasing positive attitudes towards cord blood donation and enhance women's perceptions of control over this behaviour. Targeting these constructs may be particularly relevant, given that cognition‐based interventions have been shown to be effective in other contexts, such as blood donation [35]. Although the present study did not consider whether expectant mothers were counselled by health care providers to donate their cord blood, future studies could examine whether perceptions of control and social pressure change as a result of the counselling sessions received.

For example, educational interventions and motivational interviews to decrease ambivalence and resistance towards UCB donation may be considered as means to increase intentions to donate.

The present study has some limitations that need to be acknowledged. First, despite the fact that assessing UCB donation behaviour would have been optimal, it is important to note that only 2.2% of women donates UCB in Italy donate UCB [12], similar to other countries [36, 37]. Therefore, a much larger initial sample would have been necessary to estimate the intention–behaviour pathway. Nevertheless, we know from previous studies and meta‐analyses on TPB that intentions generally range from 13.8% to 29% for different health behaviours (e.g., physical activity, diet, breastfeeding, safe sex, abstinence from drugs) [38, 39] and from 19% to 38% for blood donation behaviour [18]. Future population studies are needed to evaluate how behavioural intentions will determine actual donations in the specific context of UCB donation. Secondly, the study considered a convenience sample. Participants were expectant mothers who volunteered to be part of the study, and they may differ from mothers who were not available (i.e., a general tendency to be altruistic). Therefore, caution is needed in generalizing. Thirdly, the present research focussed on testing the prediction of intentions within one model, the TPB. Thus, the structural paths examined were those specified when following this framework. Evaluating the contribution of other models, as well as integrating the TPB model with other constructs (self‐efficacy, self‐identity, etc.) could hypothetically generate distinct results. Finally, although previous studies in the blood donation context reported that past behaviour was predictive of intentions, this variable could not be used in the present study because only 2.7% of participants had donated UCB in the past.

In conclusion, the results of the present study demonstrate the usefulness of TPB in predicting intentions to donate UCB and highlight the importance of partner and significant others in forming positive intentions to donate UCB. Unlike other studies on blood and organ donation [17, 18, 40], it appears that subjective norms play a stronger role in determining intentions in the context of UCB. This is consistent with other research on bone marrow donation [41]. These aspects, along with positive attitudes and perceived control over cord blood donation, should be considered when planning future interventions to increase donation intentions.

## CONFLICT OF INTEREST

The authors declare that they have no conflict of interest.

## Supporting information


**Appendix S1.** Supplementary information.
