# Supplementary material for: Determinants of the intention to donate umbilical cord blood in pregnant women
Source: Vox Sang. 2021 Jul 14;117(2):169–76. doi: 10.1111/vox.13179 (PMC9291455; doi:10.1111/vox.13179)
Supplement: Supplementary file 1 — Appendix S1. Supplementary information. [file VOX-117-169-s001.docx]

**Appendix 1**

**Attitudes towards UCB donation**

| *Donate the blood cord of my baby would be for me…* | | | | | | | | |
| --- | --- | --- | --- | --- | --- | --- | --- | --- |
| Useless | 1 | 2 | 3 | 4 | 5 | 6 | 7 | Useful |
| Difficult | 1 | 2 | 3 | 4 | 5 | 6 | 7 | Easy |
| Disappointing | 1 | 2 | 3 | 4 | 5 | 6 | 7 | Rewarding |
| Senseless | 1 | 2 | 3 | 4 | 5 | 6 | 7 | Sensible |
| Disadvantagenous | 1 | 2 | 3 | 4 | 5 | 6 | 7 | Advantageous |
| Unsatisfactory | 1 | 2 | 3 | 4 | 5 | 6 | 7 | Satisfactory |
| Wrong | 1 | 2 | 3 | 4 | 5 | 6 | 7 | Right |
| Stupid | 1 | 2 | 3 | 4 | 5 | 6 | 7 | Wise |
| Unnerving | 1 | 2 | 3 | 4 | 5 | 6 | 7 | Reassuring |
| Expensive | 1 | 2 | 3 | 4 | 5 | 6 | 7 | Economical |

**Subjective Norms regarding UCB donation**

*Most people who are important to me think that I should donate the cord blood of my baby*

| Completely disagree | ❒ 1 | ❒ 2 | ❒ 3 | ❒ 4 | ❒ 5 | ❒ 6 | ❒ 7 | Completely agree |
| --- | --- | --- | --- | --- | --- | --- | --- | --- |
|  |  |  |  |  |  |  |  |  |

*Most people who are important to me would approve that I donate the cord blood of my baby*

| Completely disagree | ❒ 1 | ❒ 2 | ❒ 3 | ❒ 4 | ❒ 5 | ❒ 6 | ❒ 7 | Completely agree |
| --- | --- | --- | --- | --- | --- | --- | --- | --- |

*My partner thinks that I should donate the cord blood of our baby*

| Completely disagree | ❒ 1 | ❒ 2 | ❒ 3 | ❒ 4 | ❒ 5 | ❒ 6 | ❒ 7 | Completely agree |
| --- | --- | --- | --- | --- | --- | --- | --- | --- |

*My partner would approve that I donate the cord blood of our baby*

| Completely disagree | ❒ 1 | ❒ 2 | ❒ 3 | ❒ 4 | ❒ 5 | ❒ 6 | ❒ 7 | Completely agree |
| --- | --- | --- | --- | --- | --- | --- | --- | --- |

*My partner would like me to donate the cord blood of our baby*

| Completely disagree | ❒ 1 | ❒ 2 | ❒ 3 | ❒ 4 | ❒ 5 | ❒ 6 | ❒ 7 | Completely agree |
| --- | --- | --- | --- | --- | --- | --- | --- | --- |

**Perceived Behavioural Control over UCB donation**

*Suppose you decide to donate your baby cord blood. How easy or difficult do you think it will be…?*

| Very difficult | ❒ 1 | ❒ 2 | ❒ 3 | ❒ 4 | ❒ 5 | ❒ 6 | ❒ 7 | Very easy |
| --- | --- | --- | --- | --- | --- | --- | --- | --- |

*Deciding to donate the cord blood of your baby is…?*

| Not at all up to me | ❒ 1 | ❒ 2 | ❒ 3 | ❒ 4 | ❒ 5 | ❒ 6 | ❒ 7 | completely up to me |
| --- | --- | --- | --- | --- | --- | --- | --- | --- |

*How much control do you feel you have over your decision to donate your baby’s umbilical cord blood?*

| Not at all under my control | ❒ 1 | ❒ 2 | ❒ 3 | ❒ 4 | ❒ 5 | ❒ 6 | ❒ 7 | Completely under my control |
| --- | --- | --- | --- | --- | --- | --- | --- | --- |

**Intention to donate umbilical cord blood**

*Do you intend to donate your baby’s umbilical cord blood?*

| I definitely do not | ❒ 1 | ❒ 2 | ❒ 3 | ❒ 4 | ❒ 5 | ❒ 6 | ❒ 7 | I definitely do |
| --- | --- | --- | --- | --- | --- | --- | --- | --- |

*I would like to donate my baby's umbilical cord blood*

| I definitely do not | ❒ 1 | ❒ 2 | ❒ 3 | ❒ 4 | ❒ 5 | ❒ 6 | ❒ 7 | I definitely do |
| --- | --- | --- | --- | --- | --- | --- | --- | --- |

*I will try to donate my baby's umbilical cord blood*

| I definitely do not | ❒ 1 | ❒ 2 | ❒ 3 | ❒ 4 | ❒ 5 | ❒ 6 | ❒ 7 | I definitely do |
| --- | --- | --- | --- | --- | --- | --- | --- | --- |

*How strong is your intention to donate your baby's umbilical cord blood?*

| Not strong at all | ❒ 1 | ❒ 2 | ❒ 3 | ❒ 4 | ❒ 5 | ❒ 6 | ❒ 7 | Very strong |
| --- | --- | --- | --- | --- | --- | --- | --- | --- |

*How likely are you to donate your baby's umbilical cord blood?*

| Not at all likely | ❒ 1 | ❒ 2 | ❒ 3 | ❒ 4 | ❒ 5 | ❒ 6 | ❒ 7 | Very likely |
| --- | --- | --- | --- | --- | --- | --- | --- | --- |
